# Nurses’ knowledge and practices in the face of the challenge of using the systematization of nursing care as an instrument of assistance in a first aid in Brazil

**DOI:** 10.1097/MD.0000000000011509

**Published:** 2018-08-17

**Authors:** Rosicley S. da Silva, Italla M.P. Bezerra, Carlos B.M. Monteiro, Fernando Adami, Hugo M.F. Souza, Luiz C. de Abreu

**Affiliations:** a Setor de Pós-Graduação, Pesquisa e Inovação, Faculdade de Medicina do ABC, FMABC, Santo André, São Paulo; b Laboratório de Escrita Científica da UNINORTE, Rio Branco, Acre; c Programa de Pós-Graduação em Políticas Públicas e Desenvolvimento Local. Escola Superior de Ciências da Santa Casa de Misericórdia de Vitória, EMESCAM, Vitória, ES; d Programa de Pós-graduação em Ciências da Saúde, Universidade Federal do Acre, UFAC, Rio Branco, Acre; e Escola de Artes, Ciências e Humanidades da Universidade de São Paulo, EACH-USP, São Paulo, São Paulo, Brasil.

**Keywords:** nursing diagnosis, nursing process, protocol study, qualitative research

## Abstract

Objective: To analyze the performance of nurses in the implementation of nursing care systematization (NCS). This study is a descriptive research developed from a qualitative approach. The content analysis (CA) must be developed through 3 chronological poles allowing the researcher to construct an analysis structure that corresponds to the needs of the research and the objectives of the proposed research; The chronological poles of CA are described as: Phase 1—preanalysis, phase 2—exploration of the material: phase 3—treatment of the results obtained and interpretation. Only a semistructured interview will be conducted with the research subjects who meet the inclusion criteria of the study, preserving the identity of the individuals and guaranteeing the right to quit the research at any time during the interview. The Research Ethics Committee of Hospital of the clinics of Acre, Brazil (Amazon region) under the opinion no. 1.460.960 approved this protocol. The clinical protocol was registered in the “Brazilian Registry of Clinical Trials” validated by the World Health Organization, and received clinical trials “RBR-882rg2.”

## 1 Introduction

The activities related to the care offered by a health team have as partners the nurses who work in the coordination, supervision, and specialized technical care.^[1]^

The activities of nurses are developed in an integrated and concomitant way; the large dimension of nursing care is possible because the nurse has the vision and knowledge allied to a leader's posture and collaborative behavior.^[1]^

In order to develop assistance activities with quality, the technology allied to the nursing care systematization (NCS) directs nurses to an evidence-based practice, valuing the planning capacity necessary for daily practice.^[1–2]^

The NCS is the instrument that provides the use of scientific principles and a work methodology that promotes the interrelationship of actions, favoring the assistance rendered to the human being, contributing to the quality of emergency care.^[2–4]^

Also defined as a systematic and dynamic way of providing nursing care, using 5 interrelated stages: nursing history (NH), nursing diagnosis (ND), nursing planning (NP), nursing implementation (NI), and nursing evaluation (NE).^[5]^

Reinforcing the importance and necessity of systematizing nursing care, Federal Nursing Council (COFEN) Resolution 358/2009, in its article 1, states that the implementation of NCS must take place in every public and private health institution.^[5]^

According to Vaz et al,^[6]^ the NCS assists in building the quality of health care and also in building theoretical and scientific knowledge based on the best clinical practices. For its development, the professional needs to master the 5 stages of this work process; however, problems in the planning and optimization of the human resources can lead the nurse to commit flaws in the service, damaging the final result of the assistance rendered.^[2,3,7]^

According to Oliveira et al^[8]^ and Cruz and Almeida,^[9]^ the discourses point to a mechanized assistance with low critical clinical reasoning, with a view to fulfilling the routine tasks destined to the execution of the care, being evident the lack of adhesion of the nursing team to a plan of care focused on the patient.

In the category of difficulties in the implementation of NCS, it is pointed out that it is devalued by the nurse and by the entire nursing team; this position is based on the lack of specific knowledge of these professionals on the NCS, becoming a barrier to its execution in health institutions.^[10–12]^

The lack of scientific knowledge favors its devaluation, especially in nursing teams where there is no multiprofessional space for discussions about NCS. Thus, care planning has been in the background in many realities, once perceived as a difficult activity to be practiced on the Brazilian nursing scene, despite all the logical proposal presented by NCS.^[9,10]^

Therefore, nurses often move away from their main role, due to the need to perform bureaucratic and routine activities, causing a distance between the nurse and the patient.^[4]^

In this way, in view of the reflections presented so far, some questions are pertinent to help in the cut of the object of this study, such as: what is the nurses’ perception about NCS? How was the process of training professionals to implement this instrument? What elements can make this implementation easier or more difficult? In front of these questions, it is hypothesized that nurses do not present sufficient knowledge to develop NCS and the actions to implement it.

Considering that this instrument is part of nurses’ work, the objective of the study proposed here is to analyze the nurses’ performance in the implementation of NCS.

## 2 Methods

### 2.1 Study design

It is a descriptive research developed from a qualitative approach.

The descriptive studies, according to Gil^[13]^ have as main objective the description of characteristics of a certain population phenomenon or the establishment of relationships between variables obtained through standardized techniques of data collection, such as questionnaire and systematic observation.

To Triviños,^[14]^ descriptive studies are intended to accurately demonstrate the facts and phenomena of a reality, which requires the researcher to have a series of information about what one wishes to research, for example, population, study objectives, hypotheses, assumptions and questions of search. The descriptive study will allow to accurately present facts or phenomena of the studied reality.

The qualitative approach, according to Minayo,^[15]^ arises from the impossibility of investigating and understanding by mean of statistical data some phenomena focused on perception, intuition, and subjectivity. Through qualitative evaluation we can expose experience and common sense through the actions of understanding, interpreting and promoting the dialectics necessary for understanding.^[16]^

### 2.2 Research scenario

The implementation of this research protocol will be performed at the Hospital of Rio Branco—HUERB, an institution maintained by the state public administration, linked to the State Health Secretariat, a member of the Unified Health System (SUS). The mission of HUERB is to provide humanized care with quality and ethics, respecting sociocultural diversity to all who seek an emergency care service.

This emergency service attends clinical emergencies, surgical, and traumatic in adults and children, with back-up beds in medical clinic, treatment of acute intoxication and alcohol and other drug withdrawal, pediatric emergency, surgical centre, and service of intensive care unit. Altogether there are 204 beds distributed between beds of hospitalization, observation, and emergency.

This component of the Urgency and Emergency Network is one of the entry points for secondary level of health care and is a reference for the entire State of Acre, Brazil, and border countries, Bolivia and Peru.

### 2.3 Study participants

The public servants, nurses, who carry out assistance activities at HUERB will be part of the research participants. It is estimated 30 interviews that will be consolidated by the saturation method.

### 2.4 Inclusion criteria

Will be included in the survey, the nurses, who are at the Emergency of Hospital of Rio Branco—HUERB who agree to participate in the interview and who sign the Informed Consent Form (ICF).

### 2.5 Collection instrument of empirical material

A semistructured interview will be used for data collection with 16 guiding questions addressing aspects related to NCS knowledge and practices.

The semistructured interviews will valorise the researcher's presence and offer all possible perspectives to reach the spontaneity necessary for qualitative research. The interviews start from certain questions that are not born a priori, but from information that the researcher already has about the phenomenon that is of interest to study. In this sense, the informant will have the freedom to follow the line of his thought and his experiences within the main focus placed by the investigator.^[14]^

### 2.6 Process of saturation, corpus organization and data analysis

The organization of the chronological poles will be carried out according to the technique of content analysis (CA) through stages that will enable to describe and interpret the subjects’ speeches.^[17]^

**Figure 1:**
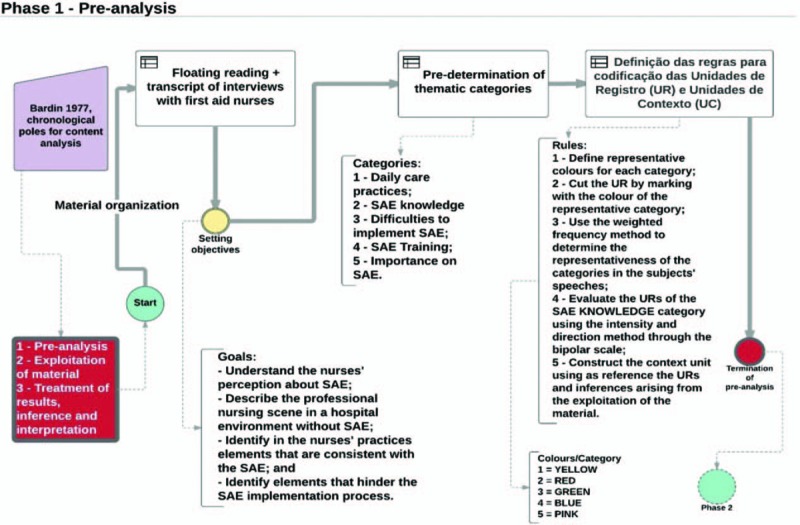
Preanalysis flowchart. ∗RU/UR—record units; ∗CU/UC—context units; ∗SAE/NCS—Nursing Care Systematization. Source: Author, 2017.

According to Bardin^[17]^ the CA must be developed through 3 chronological poles allowing the researcher to construct an analysis structure that corresponds to the needs of the research and the objectives of the proposed research; The chronological poles of CA are described as follows (Fig. 1):

- Phase 1—Pre-analysis: stage of the organization itself. In it the documents that will be submitted for analysis are selected, the hypotheses and the objectives are formulated and the rules that base the final interpretation are elaborated.
- Phase 2—Material exploration: this stage consists essentially of coding, discount, or enumeration operations, according to preformulated rules. It constitutes the identification of the units of record, units of context, and subjects that arise from the readings. In this stage, the thematic units and registration units of this study will be identified. It is emphasized that the thematic categories that were explored in this phase will be built focused on the objectives in the previous phase.
- Phase 3—Treatment of results obtained and interpretation: the gross results will be treated in a way that in the end has a meaning. At this stage, the investigator may propose inferences and advance interpretations about the intended objectives or relate to other unexpected discoveries.

For the completion of data collection (conducting interviews) the researcher will consider the process of saturation of the speeches of the subjects which consists in analyzing the empirical data that will be collected. The investigator should focus on the perception of the decrease of new information in each interview, considering the saturation point when the new interviews do not present additions or insignificant information to the research directed by the objectives of the protocol.^[18]^

Following the steps proposed by Bardin^[17]^ in the preanalysis, the researcher will transcribe the interviews that will allow the floating reading necessary for the initial organization of CORPUS (set of documents that will be exposed to the analysis) that will be analysed; Reaffirmed the objectives of the study, that are: understand the nurses’ perception about NCS; Describe the panorama of nursing professional performance in a hospital environment without NCS; Identify in nurses’ practices elements that are consistent with the NCS; and identify elements that hinder the NCS implementation process.

Based on the objectives of the study, the researcher predetermined the thematic categories named as:

1. daily practices in assistance;
2. the NCS Knowledge;
3. difficulties to implement the NCS;
4. the NCS training; and
5. the NCS importance.

Thus, to determine the end of the collection, the saturation process will be used; the researcher should consider the convergence of the respondents’ speeches considering the categories mentioned above.

For the development of the CA is oriented the codification of textual fragments called record units (RU) and subsequent construction of the called context units (CU) that provide sensing to the speeches of respondents; for the implementation of the codification, in the material exploration stage, rules were defined for the systematic development of the enumeration and codification of the registration units by the researcher. The default rules were:

Define representative colors for each category: Category 1 = Yellow; Category 2 = Red; Category 3 = Green; Category 4 = Blue; and Category 5 = Pink;

1. Cut the RU by marking the color of the representative category.
2. Use the weighted frequency method to determine the representativeness of the categories in the subjects’ speeches.
3. To evaluate the RUs of **NCS** Knowledge using the intensity and direction method through the bipolar scale;
4. Construct the CU using as reference the RUs and inferences arising from the exploration of the material.

Figure 1 presents the flowchart of the construction of the preanalysis and structuring of *CORPUS* for CA.

After all the reasoning of the rules and how the researcher will direct their analysis of content, will proceed the second stage called *exploration of the material*; to begin the rules implementation the researcher should develop a new reading of *CORPUS*.

The *CORPUS* in this research will be structured in an excel worksheet composed by the transcription of the answers of the respondents, tables with the registration of RU, CU and analytical categories according to Bardin^[17]^ technique, analytical categories and evidences identified with the analysis, registration of the weighted frequencies of the RUs by category, evaluation of the direction and intensity of the RUs for the **NCS***Knowledge* category and a synthesis of saturation/category points that determined the closure of the data collection.

In the implementation of the *CORPUS* reading, the RU/category will be cut using the colored rule predetermined in the previous step for all the speeches of the subjects interviewed.

The application of the weighted frequency will allow the correct direction of the analysis of each questioning for the thematic categories and the use of the intensity and direction for the RUs referring to the NCS Knowledge category to define the intensity of the right and wrong answers by the subjects.

The exploration of the material will enable the codification and enumeration of RUs and the construction of CUs, which is the first significant inference for the analysis of the subjects’ speech and interpretations made by the researcher. Table 1 shows an example of how the RU and CU will be organized by analytical category, which is the product of the exploration phase of the material.

**Table 1:**
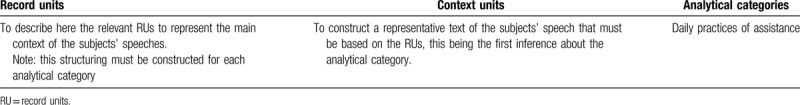
Record units, context units, and analytical categories according to the Bardin method (Rio Branco, Acre, 2017).

**Figure 2:**
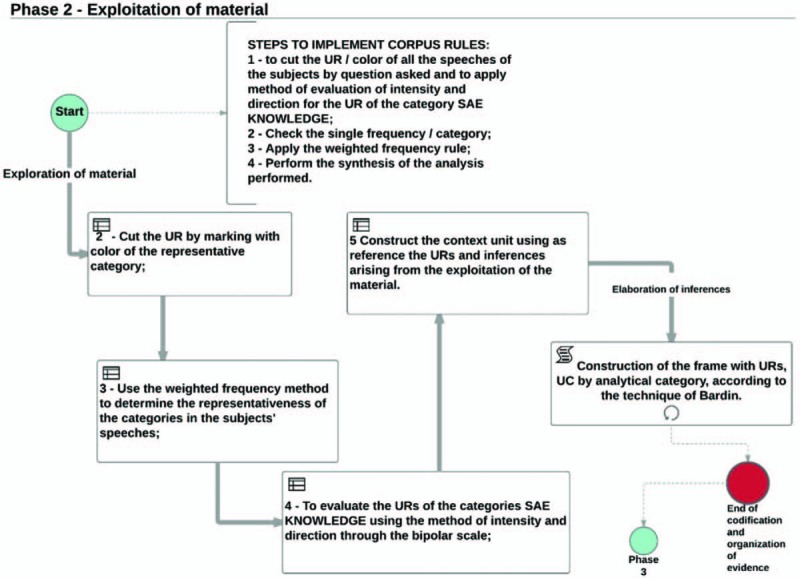
Flowchart of the material exploration step. ∗RU/UR—record units; ∗CU/UC—context units; ∗SAE/NCS—Nursing Care Systematization. Source: Author, 2017.

Figure 2 represents an example of the flowchart of the implementation of the rules described above and the beginning of the organization of the evidences that will be identified until step 2.

In the third stage, called *Treatment of the results obtained and interpretation*, a revision of the CU will be implemented, finalizing the construction of it and organizing the main evidences raised in step 2 to proceed a later interpretation of the results obtained with the exploration of the material.

**Figure 3:**
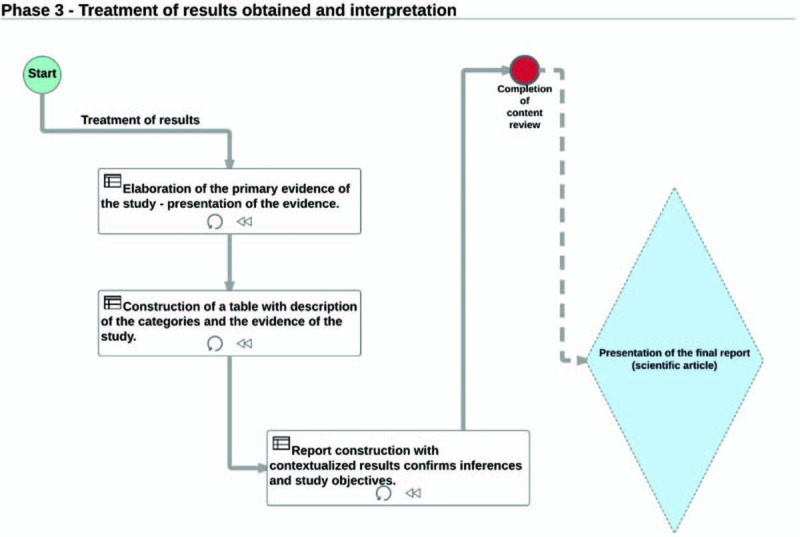
Flowchart of the step treatment of the results obtained and interpretation, Source: Author, 2017.

In this stage the construction of the table with the categories and a summary of the evidences that will be submitted to the interpretation of the researcher are going to completed and will support the construction of the final report of the results and final discussion of the research in the light of the NCS literature. Figure 3 shows the flowchart of the phase of the results treatment.

Table 2 represents the example of how the first evidence of the categories of study will be organized.

**Table 2:**
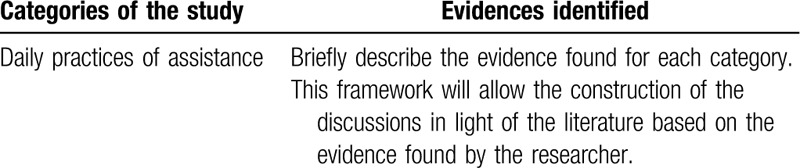
Description of study categories and evidence. Rio Branco, Acre, Brazil 2017.

### 2.7 Risks and benefits of research

The risks arising from the research will be related to the discomfort and/or embarrassment in answering some of the interview questions because it is an evaluation of the knowledge necessary for the implementation of a work tool (NCS) as well as the data collected in the interviews could be exposed.

### 2.8 Primary and secondary outcomes

As a primary outcome, nurses’ knowledge about NCS will be presented and, as a secondary outcome, the difficulties will be encountered for the implantation of NCS in an emergency hospital.

### 2.9 Ethical aspects and dissemination of research

The Research Ethics Committee of ACRE - HCA/FUNDHACRE CLINICS HOSPITAL evaluated the protocol for this research, approving on March 22, 2016 under the opinion 1,460,960, since the protocol met the requirements of Resolution 466/12, of the National Health Council/Ministry of Health, which deals with research involving human beings.^[19]^

Only the semistructured interview will be conducted with the research subjects who meet the inclusion criteria of the study, preserving the subject's identity and guaranteeing the right to quit the research at any time during the interview.

For the preparation of the final report of this research, when there is a need to quote the subject's speech, they will be identified with the letter “E” accompanied by the number representing the research subject, for example, E1, E2, successively.

## 3 Discussion

Nowadays, it is urgent to organize the nursing service in public and private units through the implementation of NCS; however, it is still necessary to awake the professionals to the need of searching for knowledge and to reduce the dichotomy between academia and professional practice.^[2,5,20–23]^

The training of the professionals does not contribute to the application of the NCS, since there is no implementation of the investigation due to lack of preparation which in fact leaves them deprived of the understanding of the real role of nursing.^[2]^

The use of the technique of CA according to the guidelines of Bardin^[17]^ will help in the interpretation of the speeches of respondents who in many cases do not believe that they are performing practices outside the standard established by the science of nursing; the interpretation of knowledge and practices without a rigorous technique could lead to subjective and superficial interpretations as to what is intended to be analyzed with this protocol of research, but as reported by Minayo^[15]^ with the support of qualitative research can be reached conclusions that are not measured by statistics.

Qualitative research is capable of incorporating the question of meaning and intentionality as inherent in acts, relationships and social structures, the latter being taken, both in its advent and in its transformations, as significant human constructions.^[17]^

Thus, the qualitative approach applies to the study of history, relationships, representations, beliefs, perceptions and opinions, the product of interpretations of what human beings do, how they live, how they build themselves, feel and think, making possible an analysis and inference that will portray the reality of the subjects’ speeches, being the best option to reach the answers of this research protocol.^[18]^

## 4 Innovation

The information analyzed by this research protocol will have the power to encourage the reflection of many nursing professionals about their daily work, by giving visibility to the importance of the NCS implementation, and to illustrate the direction of the nurses to be guided by the evidence and planning required for a good quality nursing care.

The understanding of the knowledge and practices on the NCS can reach the excellence of the assistance provided to the user of the emergency services, a reflection on the nursing work instrument and the increase of the implantation of NCS in the health care units.

## Acknowledgments

To Acre State through the Department of Health for the opportunity to qualify through the agreement n. 007/2015.

To the ABC Medical School, São Paulo, Brazil, for believing in the Acreans potential, in the search for professional qualification.

To the Federal University of Acre (UFAC) for the interinstitutional partnership, academic, and intellectual support with its faculty of counsellors.

To the Floresta campus of UFAC in Cruzeiro do Sul, Acre, Brazil, partner of this project. To all those who directly or indirectly contributed to the realization of this project.

## Author contributions

**Conceptualization:** Rosicley S. da Silva, Italla M.P. Bezerra, Hugo M.F. Souza Junior, Luiz C. de Abreu.

**Data curation:** Rosicley S. da Silva, Italla M.P. Bezerra, Fernando Adami, Luiz C. de Abreu.

**Formal analysis:** Rosicley S. da Silva, Italla M.P. Bezerra, Fernando Adami.

**Funding acquisition:** Rosicley S. da Silva, Italla M.P. Bezerra, Fernando Adami.

**Investigation:** Rosicley S. da Silva, Italla M.P. Bezerra.

**Methodology:** Rosicley S. da Silva, Italla M.P. Bezerra, Carlos B.M. Monteiro, Fernando Adami, Luiz C. de Abreu.

**Project administration:** Rosicley S. da Silva, Italla M.P. Bezerra, Carlos B.M. Monteiro, Fernando Adami, Luiz C. de Abreu.

**Resources:** Rosicley S. da Silva, Italla M.P. Bezerra, Carlos B.M. Monteiro, Luiz C. de Abreu.

**Software:** Rosicley S. da Silva, Italla M.P. Bezerra.

**Supervision:** Rosicley S. da Silva, Italla M.P. Bezerra, Hugo M.F. Souza Junior.

**Validation:** Rosicley S. da Silva, Italla M.P. Bezerra, Carlos B.M. Monteiro, Fernando Adami, Hugo M.F. Souza Junior, Luiz C de Abreu.

**Visualization:** Rosicley S. da Silva, Italla M.P. Bezerra, Fernando Adami, Hugo M.F. Souza Junior, Luiz C de Abreu.

**Writing – original draft:** Rosicley S. da Silva, Italla M.P. Bezerra, Hugo M.F. Souza Junior.

**Writing – review & editing:** Rosicley S. da Silva, Italla M.P. Bezerra, Hugo M.F. Souza Junior.
